# Does Ovarian Endometriosis Increase Oocyte Sensitivity to ICSI-Induced Mechanical Damage?

**DOI:** 10.3390/jcm10081757

**Published:** 2021-04-18

**Authors:** Carlotta Scarafia, Martina Masciovecchio, Stefano Canosa, Andrea Roberto Carosso, Gianluca Gennarelli, Alberto Revelli, Chiara Benedetto

**Affiliations:** 1 Obstetrics and Gynecology 1U, Physiopathology of Reproduction and IVF Unit, Department of Surgical Sciences, Sant’Anna Hospital, University of Torino, 10126 Turin, Italy; carlotta.scarafia@gmail.com (C.S.); s.canosa88@gmail.com (S.C.); gennarelligl@gmail.com (G.G.); chiara.benedetto@unito.it (C.B.); 2 Gynecology and Obstetrics, Department of Applied and Biotechnological Clinical Sciences, San Salvatore Hospital, University of L’Aquila, 67100 L’Aquila, Italy; martimas86@gmail.com

**Keywords:** endometrioma, ovarian endometriosis, ICSI, IVF, oocyte quality

## Abstract

Some studies have shown that ICSI obtains poorer results than conventional IVF in women with ovarian endometriosis, suggesting that oocytes could be sensitive to ICSI-induced mechanical damage. The aims of this study were to clarify (a) whether ovarian endometriosis could induce peculiar fragility in the oocyte, so that ICSI would finally result harmful, and (b) whether endometrioma removal before IVF could be advisable in order to avoid any hypothetical detrimental effect. We retrospectively studied 368 women, 203 with in situ endometrioma (128 of which underwent ICSI, 75 conventional IVF) and 164 who received laparoscopic stripping of endometrioma before ICSI. For women with in situ endometrioma, cIVF and ICSI outcome was comparable for all parameters studied, including the clinical pregnancy rate per embryo transfer (PR/ET: 31.8% vs. 39.5% in the cIVF and ICSI groups) and cumulative live birth rate per ovum pick-up (CLBR/OPU: 24.4% vs. 27.7%). ICSI outcome was similar comparing women with in situ endometrioma and women previously submitted to laparoscopic stripping of cysts (CLPR/OPU 27.7% vs. 25.3%). Our findings suggest that (a) in women with in situ endometrioma ICSI may be performed, when needed, without harming oocytes and compromising the outcome and (b) that there is no advantage in removing endometrioma before ICSI.

## 1. Introduction

It was previously reported that the presence of an endometrioma could interfere with ovarian responsiveness to controlled ovarian stimulation (COS) and render oocyte retrieval more tricky and risky [1,2,3,4,5,6,7]. Besides lowering the number of available oocytes, ovarian endometriosis was also claimed to affect oocyte quality: early studies, in fact, showed that patients with an endometrioma obtained significantly lower pregnancy and implantation rates than women with other indications for IVF when using their own oocytes, but could obtain similar results when using donor oocytes [8]. The follicular fluid of women with ovarian endometriosis was found to contain an abnormally high concentration of interleukin 6 (IL-6) and a lower content of vascular endothelial growth factor (VEGF), biochemical conditions potentially able to alter oocyte development, potentially increasing oocyte fragility and susceptibility to ICSI-induced mechanical damage [9,10]. The idea that an endometrioma could impair oocyte quality and worsen oocyte competence to fertilization led to the hypothesis that using ICSI instead of conventional IVF (cIVF) could be the way to obtain a higher fertilization rate and improve results. A study on patients aged 25–40, with ovarian endometriosis, showed that although the fertilization rate was higher inseminating sibling oocytes by ICSI with normal semen than using cIVF, the ongoing pregnancy rate after ICSI was unusually low (15.6%), suggesting induced mechanical oocyte damage [11]. Moreover, recent research by Tan et al. retrospectively compared the outcome of cIVF vs. ICSI in women with endometriosis at various stages: patients with the disease at Stage 3 and 4 (including those with endometrioma) submitted to ICSI with normal semen obtained a significantly lower pregnancy and live birth rate than those undergoing cIVF [12], suggesting that the oocytes of these patients could be peculiarly fragile and could have been harmed by microinjection. The study by Tan et al. excluded couples with a severe male factor, which are commonly found in the clinical practice and are “forced” to receive ICSI; as it is known that semen quality affects ICSI results [13], it could be argued that if ICSI would have been performed using pathological semen, the advantage of cIVF over ICSI could have resulted to be even higher. 

In the present study, we aimed at finding an answer to the following issues: (a) can endometriomas induce peculiar fragility in oocytes, so that ICSI could harm them, finally worsening the outcome? (b) can endometrioma removal before IVF avoid this hypothetical detrimental effect, and therefore be advisable before ART when ICSI is required due to a severe dyspermia?

## 2. Materials and Methods

### 2.1. Patient Population

The present study was designed as a retrospective, real-world, case-control study. Women who underwent their first cIVF/ICSI cycle between January 2010 and December 2018 at the IVF Unit of S. Anna Hospital were considered. The inclusion criteria were the following: (i) age 18–43 years; (ii) indication to infertility treatment by means of cIVF or ICSI with own eggs; (iii) ultrasonographically diagnosed ovarian endometrioma or previous laparoscopic removal of ovarian endometrioma. The exclusion criteria were the following: (i) presence of ovarian cysts other than endometriosis; (ii) previous ovarian surgery for diseases other than endometriosis; (iii) presence of endocrine disorders or pharmacological treatments potentially interfering with the ovarian responsiveness to COS or with IVF outcome. In addition, cycles involving (i) semen donation or (ii) PGT-A or PGT-M were excluded from the analysis. ICSI was performed using fresh ejaculated sperm samples when one or more of the following conditions were observed: (i) sperm concentration <5 × 10^6^/mL, (ii) total sperm motility <40% or progressive motility <32%, (iii) total motile sperm count <2 × 10^6^. cIVF was adopted for all other indications.

The presence of endometrioma was diagnosed ultrasonographically. A typical endometrioma was defined as an unilocular cyst with a regular wall and homogenously low-level echogenicity (so called ‘ground glass’ appearance) of the cyst’s content [14].

The study was approved by the local Institutional Review Board (approval n. 0038538). Since it was a retrospective analysis of routinely collected data, specific informed consent was not required.

### 2.2. IVF Procedure

Controlled ovarian stimulation (COS) was performed either with recombinant FSH (r-FSH; Gonal F. Merck, Darmstadt, Germany), human Menopausal Gonadotropin (HP-hMG; Meropur, Ferring, Germany) or r-FSH plus recombinant LH (r-LH) in a 2:1 ratio (Pergoveris, Merck, Darmstadt, Germany). Both the long and short protocol, with GnRH-agonist or antagonist, respectively, were used. The choice of the starting gonadotropin dose was based on age, BMI, antral follicle count (AFC), and AMH circulating levels. COS was monitored by serial transvaginal US and serum estradiol (E2) measurement performed every second day from stimulation day 6–7. COS continued until at least two leading follicles reached 18 mm mean diameter, when ovulation was triggered by injecting subcutaneously either 10,000 international units (IU) of hCG (Gonasi HP, IBSA, Lugano, Switzerland) or 250 mcg of r-HCG (Ovitrelle, Merck, Darmstadt Germany). Ultrasound-guided transvaginal oocyte pick-up (OPU) was performed approximately 36–37 h after hCG administration, under local anesthesia (paracervical block). Conventional IVF (cIVF) or ICSI was performed within 4 h after oocyte collection or after 2 h from cumulus cell removal, respectively. Normal fertilization was assessed controlling the presence of two pronuclei (2PN) and the extrusion of the second polar body after 16–18 h post-insemination. All cleaved embryos were morphologically evaluated under a conventional stereomicroscope, using the IMCS score by Holte [15]. Fresh embryo transfer (ET) was performed either of one or two cleavage stage embryos on day 3 or of one blastocyst on day 5, adopting an elective single embryo transfer (eSET) policy if ≥ 5 embryos were present on day 3. The remaining embryos (if any) were kept in culture until day 5/6 for cryopreservation or discharge. The selected embryos were transferred in uterus using a soft catheter (Sydney, Cook, Bloomington, IN, USA), under ultrasound guidance, applying the technique previously published by our group [15]. When ET was performed on day 5, either with fresh or thawed blastocyst, a blastocyst morphological score [16] was applied to integrate the IMCS score. The luteal phase was supported by administering 180 mg/day natural progesterone (Crinone 8, Merck, Darmstadt, Germany) for 15 days, after which serum hCG assay was performed to ascertain conception. Patients with serum hCG > 50 IU were submitted to transvaginal US examination three weeks later, in order to confirm clinical pregnancy.

### 2.3. Statistical Analysis

The following variables were collected for all enrolled patients: age, duration of infertility, BMI, basal AMH, AFC, smoke habit. For all ART treatment cycles, the following variables were registered: type of pituitary suppression (GnRH agonist or antagonist), total FSH dose, serum peak estradiol (E2), number of retrieved oocytes, Ovarian Sensitivity Index (OSI = number of retrieved oocytes/total FSH dose × 1000 [17]), number of mature (MII) oocytes, number of fertilized (2PN) oocytes, number of available embryos, proportion of top-quality embryos (IMCS score > 8/10). 

The primary outcome was the cumulative clinical pregnancy rate (CcPR) per OPU, calculated by dividing the sum of all clinical pregnancies derived from fresh and frozen ETs by the number of OPUs. Further parameters were also compared: fertilization and cleavage rates, implantation rate, cPR per OPU and per ET, cumulative live birth rate (CLBR) per OPU, twin rate and abortion rate. The fertilization rate was calculated as follows: in ICSI cycles as the ratio between normally fertilized oocytes and inseminated oocytes; in cIVF cycles as the ratio between normally fertilized oocytes and the oocytes that were found to be at the MII stage during the fertilization check. Embryos were defined as top quality when their morphology was judged ≥ 8/10 points at IMCS score (including number of blastomeres, fragmentation, symmetry, equality, nuclearity [15]) and/or grade 1 at the blastocyst score [18]. Statistical analysis was carried out using the Graphpad Prism V7 software. ANOVA, Kruskal–Wallis, or Mann–Whitney tests were used, as appropriate, after normality assessment by the Shapiro–Wilk test. Categorical variables were compared by the chi-square test. A *p*-value < 0.05 was considered statistically significant.

## 3. Results

Retrospectively checking our database, we found 203 women in which one or more ovarian endometriomas were present within the ovary at the time of IVF: some of them had a husband with severe dyspermia and were treated by ICSI (*n* = 128), whereas others had a non-male indication to IVF and underwent cIVF (*n* = 75). The two patients’ subgroups (cIVF and ICSI) were similar for the main clinical features able to influence IVF outcome (age, BMI and ovarian reserve markers), differing only for semen quality (Table 1).

As ICSI was chosen only in case of severe male factor and cIVF was performed in all other cases (including reduced ovarian reserve), ovarian reserve markers AMH and AFC were slightly higher in the ICSI group, although not significantly. 

Comparing the treatment outcome of cIVF and ICSI, no significant difference was observed in any of the considered parameters (Table 2). Reflecting the more favorable ovarian reserve markers, ovarian responsiveness to COS was slightly, but not significantly, higher in the ICSI group (mean OSI 4.5 vs. 2.7, mean number of retrieved oocytes 8.6 vs. 6.4). At the end of the in vitro culture procedure, the mean percentage of top-scored embryos available for ET or freezing was similar in the two groups (47.7% vs. 47.5%, respectively). A total number of 114 fresh embryos were replaced in utero in the cIVF group (110 on day 3 and 4 on day 5), and 205 in the ICSI group (199 on day 3 and 6 on day 5); 20 and 33 thawed blastocysts were transferred in the two groups, respectively. The final treatment outcome, including both ET with fresh embryos and ET with thawed embryos, was comparable in the cIVF and ICSI groups, indicating a similar efficacy despite the different semen quality (Table 2).

In fact, the cPR/ET was 31.8% vs. 39.5% in the cIVF and ICSI groups, the abortion rate was 25.9% and 29.3%, and the CLBR/OPU was 24.4% vs. 27.7%, respectively. These results were confirmed after controlling for the woman’s age, BMI, number of retrieved oocytes, percentage of MII oocytes, and number of transferred embryos.

In order to ascertain whether endometrioma removal before ICSI could lead to improved results, we compared the 128 patients submitted to ICSI with an in situ endometrioma (ICSI group) with 164 women who underwent laparoscopic endometrioma stripping before ICSI (LPS-ICSI group). Women of the ICSI and LPS-ICSI groups had comparable clinical characteristics for age, BMI and markers of ovarian reserve (Table 3).

In the LPS-ICSI group, 262 fresh embryos (246 on day 3 and 16 on day 5) and 35 thawed blastocysts were transferred in utero. The treatment outcome was also comparable for all the considered parameters and after controlling for the above-mentioned clinical variables, suggesting a neutral impact of previous surgery on ICSI results (Table 4).

## 4. Discussion

Large studies comparing the results of cIVF and ICSI for the treatment of non-male factor infertility showed quite clearly that ICSI cannot offer any advantage in terms of clinical outcome [19,20,21]. Nevertheless, data showing that ovarian endometriosis negatively affects oocyte quality [8,9,10] led to the idea that using ICSI instead of cIVF could be advantageous for this specific subset of patients. A study in which sibling oocytes of women with endometriosis aged 25–40 were inseminated by either cIVF or ICSI using normal semen showed a higher fertilization rate by ICSI, but a surprisingly low ongoing pregnancy rate (15.6%), raising the suspect of an ICSI-induced mechanical damage on oocytes [11]. Another recent retrospective article reported that patients with advanced endometriosis (including those with endometrioma) undergoing ICSI with normal semen had a significantly lower pregnancy and live birth rate than those undergoing cIVF [12]. As it is quite well established that semen quality affects ICSI results [13], taken together these results obtained performing ICSI with normal semen suggest that patients with an endometrioma could have a peculiar oocyte fragility and could be disadvantaged by microinjection-related trauma.

In the present study, we aimed at clarifying whether ICSI could be detrimental for oocytes of women with an endometrioma and also, as a consequence, whether removing the endometrioma before ICSI could be advisable to avoid its negative impact on oocyte quality. We compared herein ICSI vs. cIVF in a population of 203 women with in situ endometrioma at the time of IVF, who received either ICSI (*n* = 128) or cIVF (*n* = 75) and whose basal clinical features differed only for semen quality (significantly worse in the ICSI group). We observed comparable results in terms of fertilization rate, availability of top-quality embryos, clinical pregnancies and live births, even after correction for age, BMI, number of available mature oocytes and number of transferred embryos. Indeed ICSI, as expected, could overcome the negative impact of severe male factor on fertilization and, on the other side, did not seem to harm oocytes, finally allowing us to obtain a CLBR comparable to the one obtained by couples with normal semen undergoing cIVF. 

Our findings are different from those of previously published studies [8,9,10,11,12], despite the fact that they were performed on patient populations with clinical characteristics comparable to those observed in our population. The inclusion of a higher rate of women with more severe forms of endometriosis (higher endometriosis score) in the previously studied cohorts cannot be excluded, and could potentially explain the discrepancy in the observed results. On the other side, however, the putative detrimental effect of ovarian endometriosis on oocyte quality was always linked to the intra-ovarian effect of potentially toxic substances such as growth factors and interleukins, matrix metallo-proteinases, catalytic iron, and lipid peroxide [22]. The presence of endometriosis in other pelvic areas, of adhesions or deep endometriotic nodules is probably unlikely to lead to more serious oocyte damage. Indeed, the endometriosis classification systems continue to attract criticisms because of the lack of ability in predicting fertility prognosis, suggesting a limited relationship between the disease score and the biological aspects of conception [23].

We also compared the efficacy of ICSI for women with in situ endometrioma (*n* = 128) vs. women who underwent laparoscopic removal of endometrioma before IVF (*n* = 164), observing no significant difference in treatment outcome, including the cPR/ET and the CLBR. This finding is in line with what published in systematic reviews, reporting that the surgical removal of an endometrioma prior to ICSI does not offer any advantage, implying, on the other side, an increased risk of cycle cancellation and decreased oocyte yield [6,24]. Indeed, scientific evidence shows quite convincingly that endometriomas, especially those <4 cm mean diameter, should not be removed before ART [3,25,26], unless pain is a significant concern or the sonographic aspect of the cyst is not reassuring [27]. Interestingly enough, and in contrast to what was reported in other studies [28], we observed a comparable ovarian response to COS in operated and non-operated women, who obtained a similar number of oocytes with a similar responsiveness to gonadotropins (OSI), probably as a consequence of the extremely conservative attitude of surgeons operating ovarian endometriosis at our institution.

In conclusion, we report herein that (a) in women with in situ endometrioma ICSI may be performed in case of severe male factor without inducing apparent damage over oocytes and without compromising the treatment outcome and that (b) there is no advantage in removing the endometrioma before performing ICSI, when ICSI is needed due to severe dyspermia.

## Figures and Tables

**Table 1:** Clinical characteristics of 203 patients who underwent ART treatment in the presence of endometrioma, either conventional IVF (cIVF; *n* = 75) or ICSI (*n* = 128). No statistically significant difference in the baseline clinical features was observed between groups.

Clinical Characteristics	cIVF (*n* = 75)	ICSI (*n* = 128)	*p*
Age (years)	36.0 ± 3.7	35.9 ± 3.7	Ns
BMI (Kg/m^2^)	22.1 ± 2.9	21.3 ± 3.2	Ns
AMH (ng/mL)	2.0 ± 2.5	2.8 ± 3.1	Ns
AFC	9.1 ± 5.1	12.1 ± 7.5	Ns
Duration of infertility (years)	3.2 ± 1.8	2.4 ± 1.6	Ns
Smoke (%)	13	23	Ns

ICSI was performed in case of severe male factor, cIVF in all other cases, BMI: body mass index, AFC: antral follicle count, Ns: not significant.

**Table 2:** Outcome of 203 assisted reproduction cycles of patients with ovarian endometriosis undergoing cIVF or ICSI. All differences were not significant.

Outcome	cIVF (*n* = 75)	ICSI (*n* = 128)	*p*
Stimulation protocol (%) GnRH-agonist GnRH-antagonist	80 20	70 30	Ns
Total FSH dose (IU)	2909 ± 1052	2608 ± 1300	Ns
Serum E2 peak (pg/mL)	1733 ± 1092	1023 ± 1171	Ns
OSI	2.7 ± 2.7	4.5 ± 4.9	Ns
Retrieved oocytes	6.4 ± 4.7	8.6 ± 5.6	Ns
Mature (MII) oocytes (%)	85.0 ± 20.2	79.5 ± 18.5	Ns	
Fertilization rate (%)	73.2 ± 31.6	68.5 ± 25.0	Ns
Cleavage rate (%)	88.2 ± 28.3	95.4 ± 15.1	Ns
Top-quality embryos (%) Frozen blastocysts	47.5 ± 38.8 0.5 ± 0.9	47.7 ±39.3 0.7 ± 1.4	Ns
Implantation rate (%)	23.6	28.0	Ns
Clinical PR/OPU (%)	28.0	36.7	Ns
Clinical PR/ET (%)	31.8	39.5	Ns
Cumulative PR/OPU (%)	32.9	39.2	Ns
Twin rate (%)	11.1	17.2	Ns
Abortion rate (%)	25.9	29.3	Ns
Cumulative LBR/OPU (%)	24.4	27.7	Ns

ICSI was performed in case of severe male factor, cIVF in all other cases.

**Table 3:** Clinical characteristics of patients who underwent ICSI in the presence of endometrioma ICSI (*n* = 128) or were previously operated on by having their endometrioma laparoscopically removed before ICSI (*n* = 165). No statistically significant difference in the baseline clinical features was observed between groups.

Clinical Characteristics	ICSI * (*n* = 128)	LPS-ICSI (*n* = 164)	*p*
Age (years)	35.9 ± 3.7	36.2 ± 3.8	Ns
BMI(Kg/m^2^)	21.3 ± 3.2	22.2 ± 3.5	Ns
AMH (ng/mL)	2.8 ± 3.1	2.4 ± 3.4	Ns
AFC	12.1 ± 7.5	11.8 ± 7.3	Ns
Duration of infertility (years)	2.4 ± 1.6	3.4 ± 2.1	Ns
Smoke (%)	23	15	Ns

* ICSI was performed in case of severe male factor, LPS-ICSI: laparoscopic endometrioma stripping before ICSI.

**Table 4:** Outcome of ICSI in patients with ovarian endometrioma (*n* = 128) or patients previously operated on by having their endometrioma laparoscopically removed before ICSI (*n* = 164). ICSI was performed in case of severe male factor. No statistically significant difference in the baseline clinical features was observed between groups.

Outcome	ICSI (*n* = 128)	LPS-ICSI (*n* = 164) p	*p*
Stimulation protocol (%) GnRH-agonist GnRH-antagonist	70 30	76 24	Ns
Total FSH dose (IU)	2608 ± 1300	1879.8 ± 1134.1	Ns
Serum E2 peak (pg/mL)	1023 ± 1171	1815.8 ± 1131.9	Ns
OSI	4.5 ± 4.9	3.3 ± 2.7	Ns
Retrieved oocytes	8.6 ± 5.6	7.4 ± 3.9	Ns
Mature (MII) oocytes (%)	79.5 ± 18.5	79.8 ± 20.1	Ns
Fertilization rate (%)	68.5 ± 25.0	67.9 ± 27.1	Ns
Cleavage rate (%)	95.4 ± 15.1	94.7 ± 18.7	Ns
Top-quality embryos (%) Frozen blastocysts	47.7 ± 39.3 0.7 ± 1.4	48.6 ± 37.0 0.6 ± 1.3	Ns
Implantation rate (%)	28	17.5	Ns
Clinical PR/OPU (%)	36.7	32.9	Ns
Clinical PR/ET (%)	39.5	34.7	Ns
Cumulative PR/OPU (%)	39.2	36.1	Ns
Twin rate (%)	17.2	9	Ns
Abortion rate (%)	29.3	31.3	Ns
Cumulative LBR/OPU (%)	27.7	25.3	Ns

ICSI was performed in case of severe male factor.

## Data Availability

The data presented in this study are available on request from the corresponding author. The data are not publicly available due to Department restriction.
